# Understanding AI in Healthcare: Perspectives of Future Healthcare Professionals

**DOI:** 10.7759/cureus.66285

**Published:** 2024-08-06

**Authors:** Smita R Sorte, Alka Rawekar, Sachin B Rathod

**Affiliations:** 1 Physiology, All India Institute of Medical Sciences, Nagpur, Nagpur, IND; 2 Physiology, Jawaharlal Nehru Medical College, Datta Meghe Institute of Medical Sciences (Deemed to be University), Wardha, IND

**Keywords:** undergraduate, medical students, integration, indian medical graduate, curriculum, medical education, artificial intelligence

## Abstract

Introduction

The current medical curriculum lacks comprehensive artificial intelligence (AI)-focused training, potentially impacting future healthcare delivery. This study addresses the critical gap in AI training within medical education, particularly in India, by assessing medical students' awareness, perceptions, readiness, confidence, and ethical considerations regarding AI in healthcare. Our findings underscore the necessity of integrating AI competencies into medical education to prepare future healthcare professionals for an AI-driven landscape.

Method

After obtaining ethics approval, we conducted a cross-sectional study on Bachelor of Medicine and Bachelor of Surgery (MBBS) students from the 2019-2023 batch. An exploratory survey using a validated questionnaire was employed to obtain medical students' current understanding and awareness of artificial intelligence (AI) in healthcare, perceptions, readiness, confidence, and ethical considerations in utilizing AI technologies in clinical practice.

Results

The survey received 217 responses from 2019-2023 MBBS students. We found a mean percentage of awareness score of 44.74%, a mean percentage perception score of 68.96%, a mean percentage readiness score of 91.32%, a mean percentage confidence score of 58.48%, and a mean percentage ethics importance score of 69.27%.

Males had higher awareness, confidence, and readiness scores. Conversely, females scored slightly higher in perception and the importance of ethics consideration, although not statistically significant. Junior batches outperform senior batches in perception, confidence, and readiness scores; in contrast, the awareness and ethics importance scores do not show significant differences between the two groups.

Conclusion

Our study indicates a generally positive outlook toward AI's potential to enhance healthcare delivery and patient outcomes. The study suggests a strong inclination toward further education and practical training focused on AI in healthcare, considering a solid recognition of the significance of ethical implications related to AI in healthcare. These findings highlight the importance of fostering AI literacy within medical education curricula and underscore the necessity for ongoing evaluation and adaptation to ensure that future healthcare professionals are equipped to navigate the complexities of AI in healthcare delivery while upholding ethical standards.

## Introduction

Artificial intelligence (AI) technologies such as machine learning and deep learning will revolutionize healthcare, so equipping future doctors with knowledge of these tools is critical. AI enhances diagnostic accuracy by analyzing extensive medical data and identifying patterns that may elude human detection [1]. It also enables the development of personalized treatment plans and improves operational efficiency by automating administrative tasks [2]. Furthermore, AI expands research capabilities, facilitates continuous patient monitoring, and encourages interdisciplinary collaboration [3].

Educating medical students about AI ensures that they are well-prepared to navigate and leverage technological advancements, leading to better patient outcomes and more efficient healthcare delivery. Understanding AI's ethical and practical limitations is crucial, enabling future physicians to make informed and responsible decisions [4]. Therefore, integrating AI into medical education is vital for preparing the next generation of healthcare professionals.

Problem identification

Looking at the pace of development in AI technology, these tools are expected to be an integral part of clinical workflows when medical students complete postgraduate studies. An important issue in today's medical education system is students' insufficient training in AI [5]. This knowledge gap may translate to inferior patient care and a less efficient healthcare system. The future generations of healthcare professionals should ideally be trained in the strengths and weaknesses of these technologies [6]. In India, the medical undergraduate curriculum for medical graduates does not include any concept of AI, which adds to the problem.

Gaps in current knowledge

The Carle Illinois College of Medicine in the USA, Queen's University in the UK, and the University of Toronto in Canada have introduced AI-focused courses [7]. Professional bodies such as the Association of American Medical Colleges and the Royal College of Physicians and Surgeons of Canada advocate for AI training, emphasizing data sourcing, protection, ethics, and critical evaluation [7].

Despite significant interest, comprehensive frameworks for AI curricula in undergraduate medical education are lacking, particularly in India, where the prevalence of AI training among medical students needs to be explored. Collaborative curriculum development and understanding students' perceptions of AI are essential to addressing this educational gap and preparing future healthcare professionals.

Bridging the gap

Our study aimed to bridge the gap between advancements in AI and medical education. Through an exploratory survey, we assessed students' awareness of AI concepts, perception of its application in healthcare, readiness and confidence to include AI competencies in their training, and perception of ethical considerations in using AI. The findings highlight the anticipation of AI's widespread use in healthcare, underscoring the need for a curriculum that aligns with students' expectations and technological trends. This research is crucial for developing a relevant and responsive medical curriculum. Ultimately, it prepares future healthcare professionals for an AI-driven healthcare landscape.

## Materials and methods

We obtained research cell approval (protocol number: 2024-019) on March 14, 2024, following All India Institute of Medical Sciences (AIIMS), Nagpur, Institutional Ethics Committee approval (approval number: IEC/Pharmac/2024/808, dated 03/04/2024). The study was conducted in three months (April-June 2024).

We conducted a cross-sectional study on Bachelor of Medicine and Bachelor of Surgery (MBBS) students from the 2019-2023 batch at AIIMS, Nagpur. An exploratory survey using a validated questionnaire was employed to gauge medical students' current understanding and awareness of AI in healthcare and their perception, readiness, and confidence in utilizing AI technologies, along with ethical considerations in clinical practice.

Questions were developed in English after reviewing articles on integrating AI into undergraduate medical education and prior surveys on medical student attitudes toward AI [8-12]. The questionnaire underwent pilot testing with 20 medical students and achieved a Cronbach's alpha score of 0.812 for reliability.

Questionnaires

Initially, a definition was provided to assist those unfamiliar with AI terminology.

Section A: Participant Information and Consent

This section gathers basic demographic information about the participants and obtains their consent to participate in the study. It includes fields for the participant's name, age, gender, year of study (1st, 2nd, 3rd, 4th, or Interns), and batch (2019, 2020, 2021, 2022, and 2023). Participants were also informed about the research study.

Section B: Questions 1a-5a

This section explores participants' present experiences and interests in AI education and healthcare training. The questions are designed to assess whether the participants have received any formal AI education, their interactions with AI-based healthcare tools, their interest in further AI education, and their opinions on including practical AI training and ethical considerations in the undergraduate medical curriculum. The response options for these questions were "Yes," "Not sure," and "No."

Section C: Questions 1b-12b

This section delves deeper into the participants' perceptions of AI in healthcare. It includes a series of questions with a 5-point Likert scale to evaluate various aspects such as familiarity with AI concepts, understanding of AI technologies (machine learning and deep learning), awareness of AI applications, confidence in working with AI tools, perceptions of AI's impact on healthcare, awareness of ethical considerations, and the perceived importance of integrating AI advancements into the medical curriculum (Appendices).

The survey was administered electronically via Google Forms (Google, Inc., Mountain View, CA), with invitations circulated through the WhatsApp (Meta, Menlo Park, CA) group of the MBBS batches, allowing a one-week timeframe for completion.

Data segregation and analysis

The questionnaires were carefully structured and segregated into five domains to analyze various aspects of medical students' perspectives on artificial intelligence (AI) in healthcare. The five domains included awareness of AI and its application in healthcare, perception of AI and its application in healthcare, readiness to undergo training in AI, confidence, and ethical considerations.

Questions with response options "No," "Not sure," and "Yes" were scored 1, 2, and 3, respectively. Questions with five response options were scored from 1 to 5, where 1 represented the lowest grade (e.g., Not Familiar, Very Limited, and No Impact) and 5 represented the highest grade (e.g., Very Familiar, Very Good, and Very Significant Impact). The mean score for each question was calculated to determine the participants' average response. For each domain, the mean scores of all related questions were summed to obtain a total score, which was then converted into a percentage for easy interpretation and comparison across domains.

In the awareness of AI and its application in healthcare, questions assessed participants' basic understanding of AI, awareness of AI applications, and familiarity with AI concepts in healthcare. The perception of AI and its application in the healthcare domain evaluated participants' views on the potential impact of AI on healthcare delivery and patient outcomes, as well as their perception of AI's role in the future of medicine. The readiness to undergo training in the AI domain explored participants' interest in further education and training on AI in healthcare, including their willingness to incorporate practical AI training into their curriculum. The confidence domain assessed participants' confidence in working with AI tools and technologies and in utilizing AI technologies in their future medical practice after formal training. Lastly, the ethical considerations domain gauged participants' awareness of ethical issues related to AI in healthcare and their views on the importance of ethical training in the undergraduate medical curriculum.

This comprehensive analysis provided valuable insights into the current state of AI awareness, perceptions, readiness for training, confidence, and ethical considerations among medical students. The percentage scores for each domain facilitated a clear and comparative understanding of these aspects, highlighting areas where further education and training are most needed.

Data analysis

Data were entered in Microsoft Excel (Microsoft Corp., Redmond, WA), and data analysis was performed using Jamovi open statistical software version 2.3.28 (released in 2024). Descriptive statistics were applied, presenting the results in numbers and percentages. The mean score for each question was calculated to determine the participants' average response. Some questions in the questionnaire were scored on a 3-point scale, while others were on a 5-point scale. As a result, the total scores for each domain were not directly comparable. We converted these scores into percentages to facilitate a clearer and more meaningful comparison across different domains. This normalization allowed for an easier and more intuitive understanding of the results. The Mann-Whitney U test assessed independent group differences in scores, with p-values below 0.05 deemed statistically significant.

## Results

**Figure 1:**
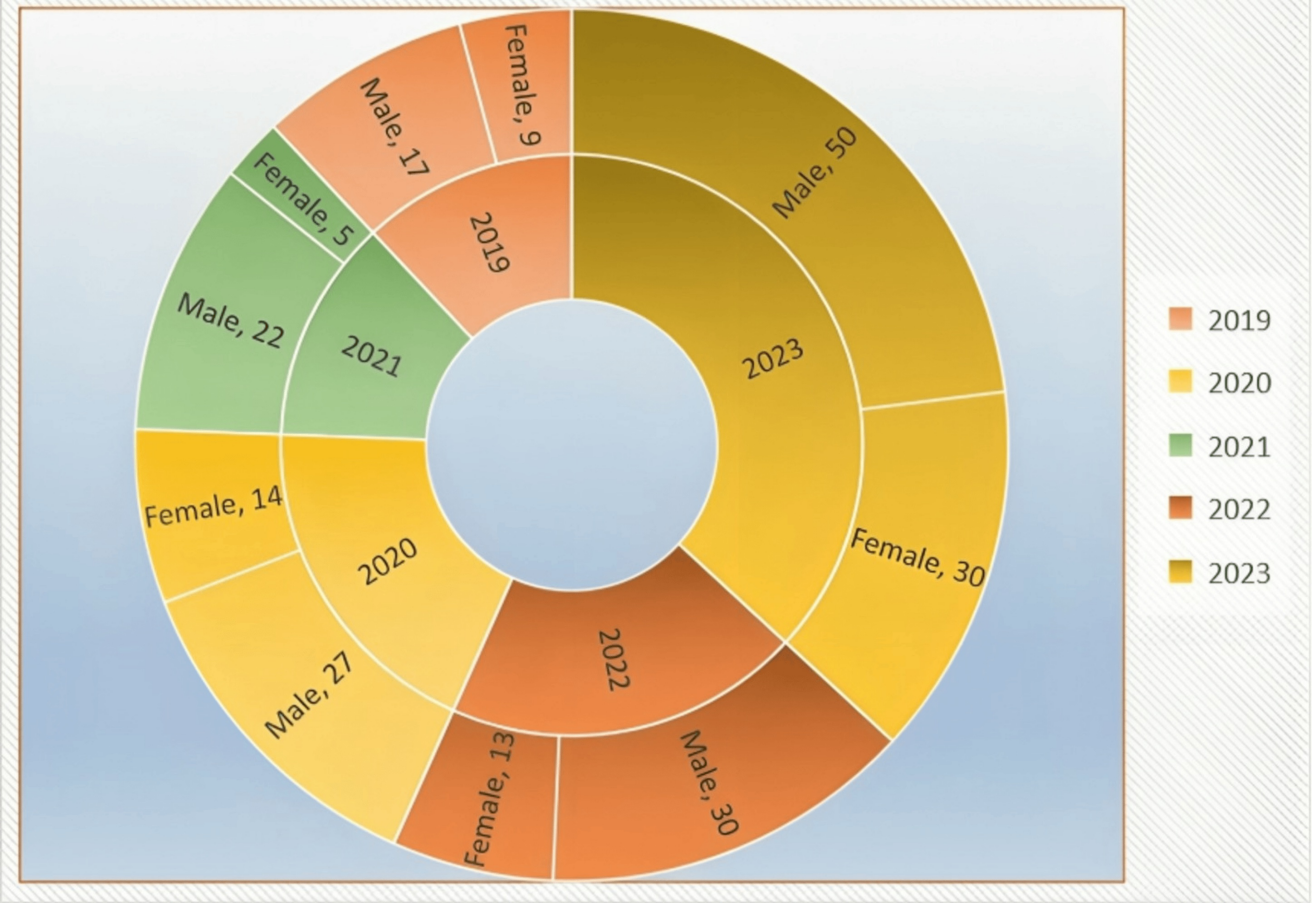
Batch and gender distribution

The survey received responses from 219 medical students spanning five years of MBBS batches. After excluding two students who did not consent, 217 valid responses were analyzed. Of the respondents, 71 (32.71%) were female and 146 (67.28%) were male. Most participants were first-year MBBS students from the 2023 batch, making up 80 (36.9%) of the sample. This was followed by students from the 2022 batch with 43 (19.8%), the 2020 batch with 41 (18.9%), the 2021 batch with 27 (12.4%), and the 2019 batch with 26 (12%) (Figure 1). 

Table 1 illustrates respondents' awareness of AI and its application in healthcare, with a mean percentage awareness score of 44.74%, indicating a moderate level of understanding across various aspects such as understanding of AI, familiarity with AI concepts, and awareness of AI applications in medicine.

**Table 1:** Scores of awareness of AI and its application in healthcare SD: standard deviation, ML: machine learning, DL: deep learning, AI: artificial intelligence

Question	Minimum points	Maximum points	Mean	SD
How would you rate your basic understanding of AI (ML and DL)?	1	5	2.15	1.096
Have you received any formal education or training on AI in healthcare?	1	3	1.16	0.521
How would you rate your awareness of AI applications?	1	5	2.52	0.923
Have you personally used or interacted with any AI-based healthcare tools or systems?	1	3	1.52	0.839
How would you rate your general familiarity with the concept of AI in healthcare?	1	5	2.27	1.043
How would you rate your awareness of the use of AI in medicine?	1	5	2.02	0.930
Awareness total score	1	26	11.63	3.486
Percentage awareness total score	1	100	44.74	13.407

Table 2 showcases respondents' perceptions of AI's role in healthcare, with a mean percentage perception score of 68.96%, indicating a generally positive outlook toward AI's potential to enhance healthcare delivery and patient outcomes. Participants also strongly believe in AI's significant impact on future medical practice. However, there is a moderate understanding of AI limitations, and respondents acknowledge the importance of integrating AI advancements into medical school curricula to keep pace with technological advancements.

**Table 2:** Score for perception of AI and its application in healthcare SD: standard deviation, AI: artificial intelligence

Question	Minimum points	Maximum points	Mean	SD
How do you perceive the role of AI in improving healthcare delivery and patient outcomes?	1	5	3.56	0.906
Do you believe that AI will significantly impact the future practice of medicine?	1	5	3.96	0.804
How would you rate your understanding regarding the limitations of AI?	1	5	2.52	0.958
How important do you think it is for medical schools to keep up with advancements in AI and update their curriculum accordingly?	1	5	3.75	1.012
Perception total score	1	20	13.79	2.394
Percentage perception total score	1	100	68.96	11.969

Table 3 indicates respondents' readiness to undergo training in AI, with a mean percentage readiness score of 91.32%. This high score suggests a strong inclination toward further education and practical training focused on AI in healthcare, highlighting the perceived importance of integrating AI education into undergraduate medical programs.

**Table 3:** Score for readiness to undergo training in AI SD: standard deviation, AI: artificial intelligence

Question	Minimum points	Maximum points	Mean	SD
Would you be interested in further education or undergraduate training specifically focused on AI in healthcare?	1	3	2.77	0.571
Do you think practical training with AI tools and systems should be part of undergraduate medical education?	1	3	2.71	0.634
Readiness total score	1	6	5.48	1.046
Percentage readiness total score	1	100	91.32	17.427

Table 4 depicts respondents' confidence in working with AI applications in healthcare, with a mean percentage confidence score of 58.48%. This moderate score suggests a moderate confidence level in utilizing AI technologies after obtaining formal training, indicating potential room for improvement in self-assurance regarding working alongside AI tools in medical practice.

**Table 4:** Score for confidence to work with AI applications in healthcare SD: standard deviation, AI: artificial intelligence

Question	Minimum points	Maximum points	Mean	SD
How would you rate your confidence in working alongside AI tools?	1	5	2.63	1.08
How confident are you in your ability to understand and utilize AI technologies in your future medical practice after obtaining formal training?	1	5	3.22	1.13
Confidence total score	1	10	5.85	1.91
Percentage confidence total score	1	100	58.48	19.12

Table 5 showcases respondents' considerations of ethics when working with AI applications in healthcare, with a mean percentage ethics importance score of 69.27%. This indicates a strong recognition of the significance of ethical implications related to AI in healthcare and suggests a consensus among respondents regarding the importance of integrating education and training on ethical considerations into undergraduate medical education.

**Table 5:** Score of ethics consideration when working with AI application in healthcare SD: standard deviation, AI: artificial intelligence

Question	Minimum points	Maximum points	Mean	SD
How aware are you of the ethical considerations related to the use of AI in healthcare?	1	5	2.30	1.084
How important do you think it is for medical professionals to consider ethical implications when using AI in healthcare?	1	5	3.96	0.976
Do you believe that education and training on ethical considerations of AI should be included in undergraduate medical education?	1	3	2.74	0.601
Ethics importance score	1	13	9.00	1.733
Ethics importance score in percentage	1	100	69.27	13.334

Table 6 compares scores between male and female medical students using the Mann-Whitney U test. Specifically, males had higher awareness (45.5% versus 43.1%), confidence (59.7% versus 56.1%), and readiness (91% versus 92%) scores. Conversely, females scored slightly higher in perception (68.9% versus 69%) and the importance of ethics (70.3% versus 68.8%). Despite these variations, the p-values indicated no significant gender differences in these areas.

**Table 6:** Mean score percentages between male and female medical students across various variables using the Mann-Whitney U test SD: standard deviation

Variables	Males (n = 146)		Females (n = 71)		Independent T-test	
	Mean	SD	Mean	SD	Statistics	(Mann-Whitney U) P
Awareness score percentage	45.5	13.8	43.1	12.6	4576	0.161
Perception score in percentage	69.0	11.8	68.9	12.4	5090	0.830
Confidence score in percentage	59.7	19.2	56.1	18.9	4560	0.146
Readiness score in percentage	91.0	17.8	92.0	16.6	5000	0.581
Ethics importance score in percentage	68.8	13.6	70.3	12.9	4785	0.350

**Figure 2:**
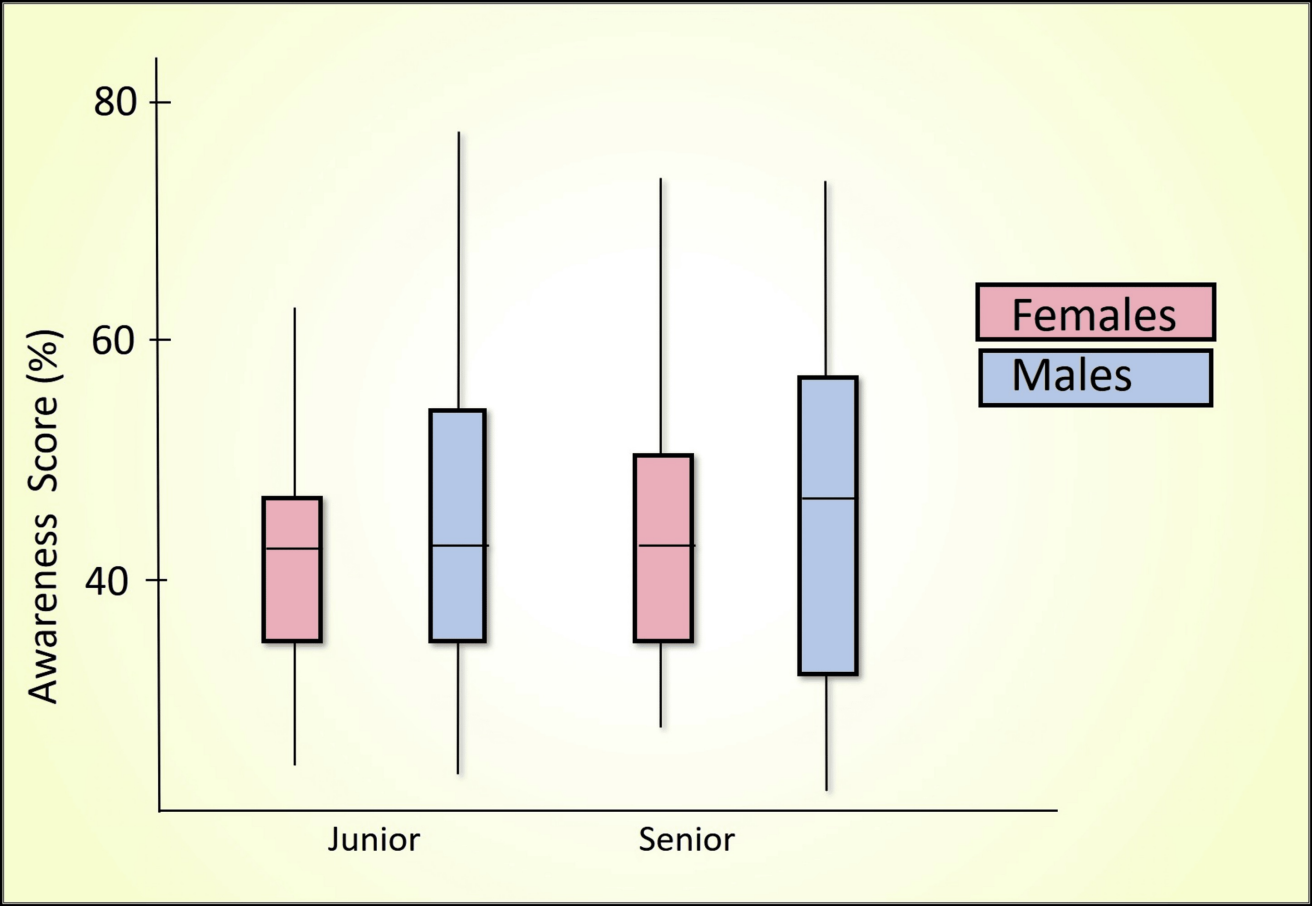
Awareness score (%) comparison by batch and gender

**Figure 3:**
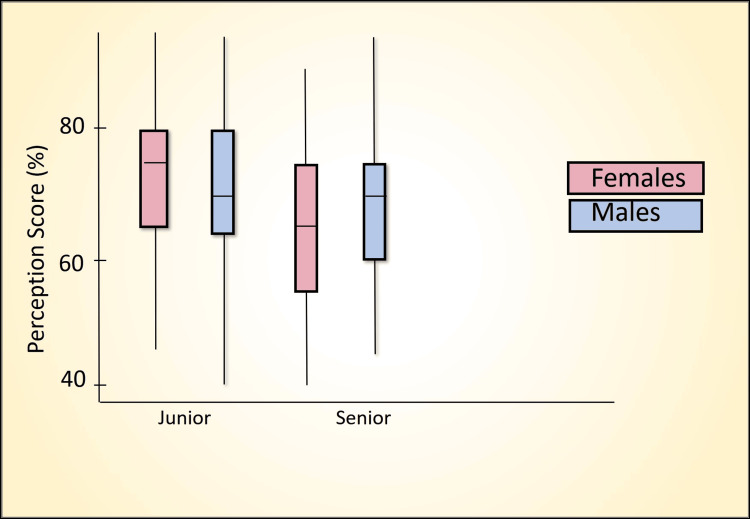
Perception score (%) comparison by batch and gender

**Figure 4:**
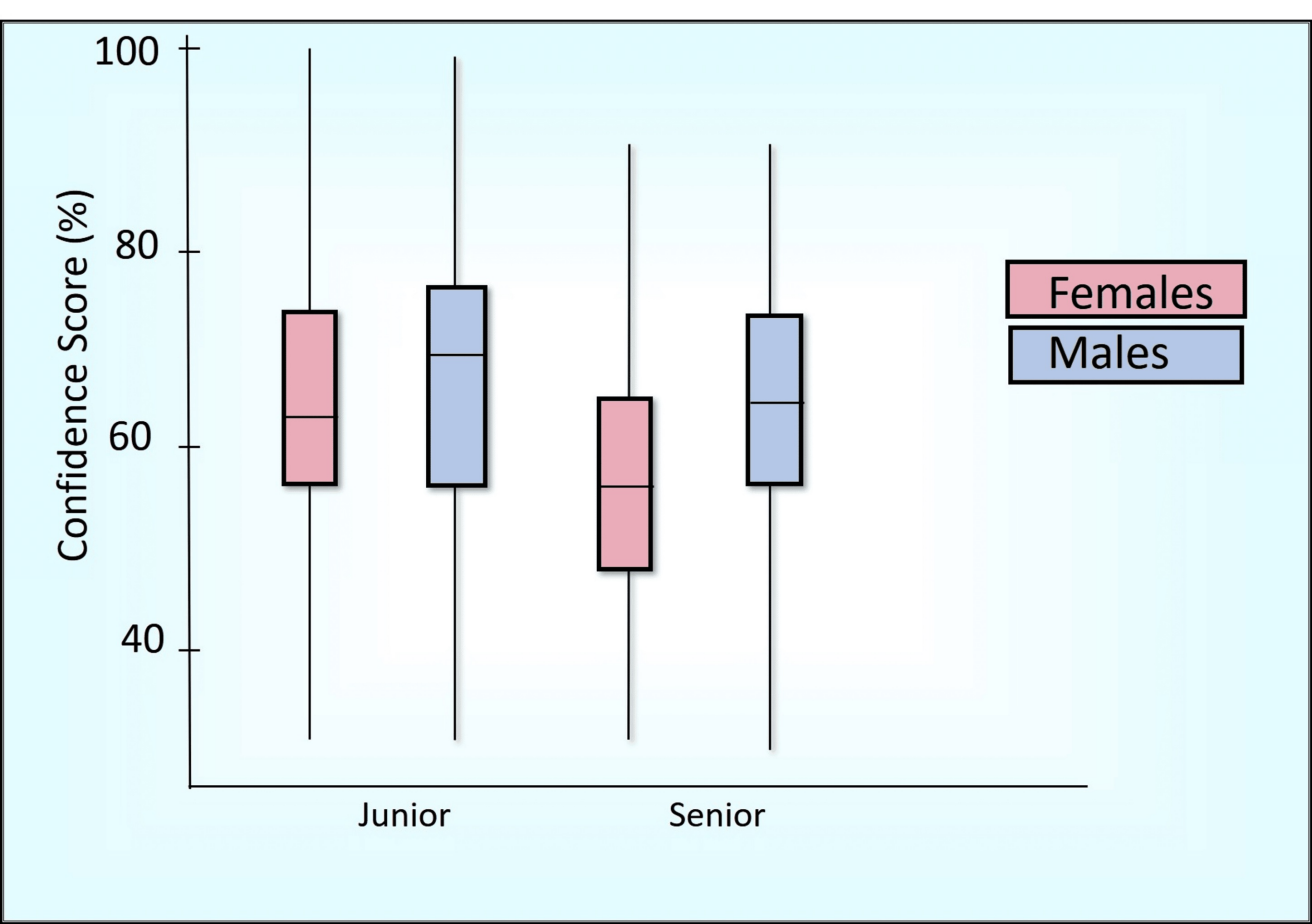
Confidence score (%) comparison by batch and gender

**Figure 5:**
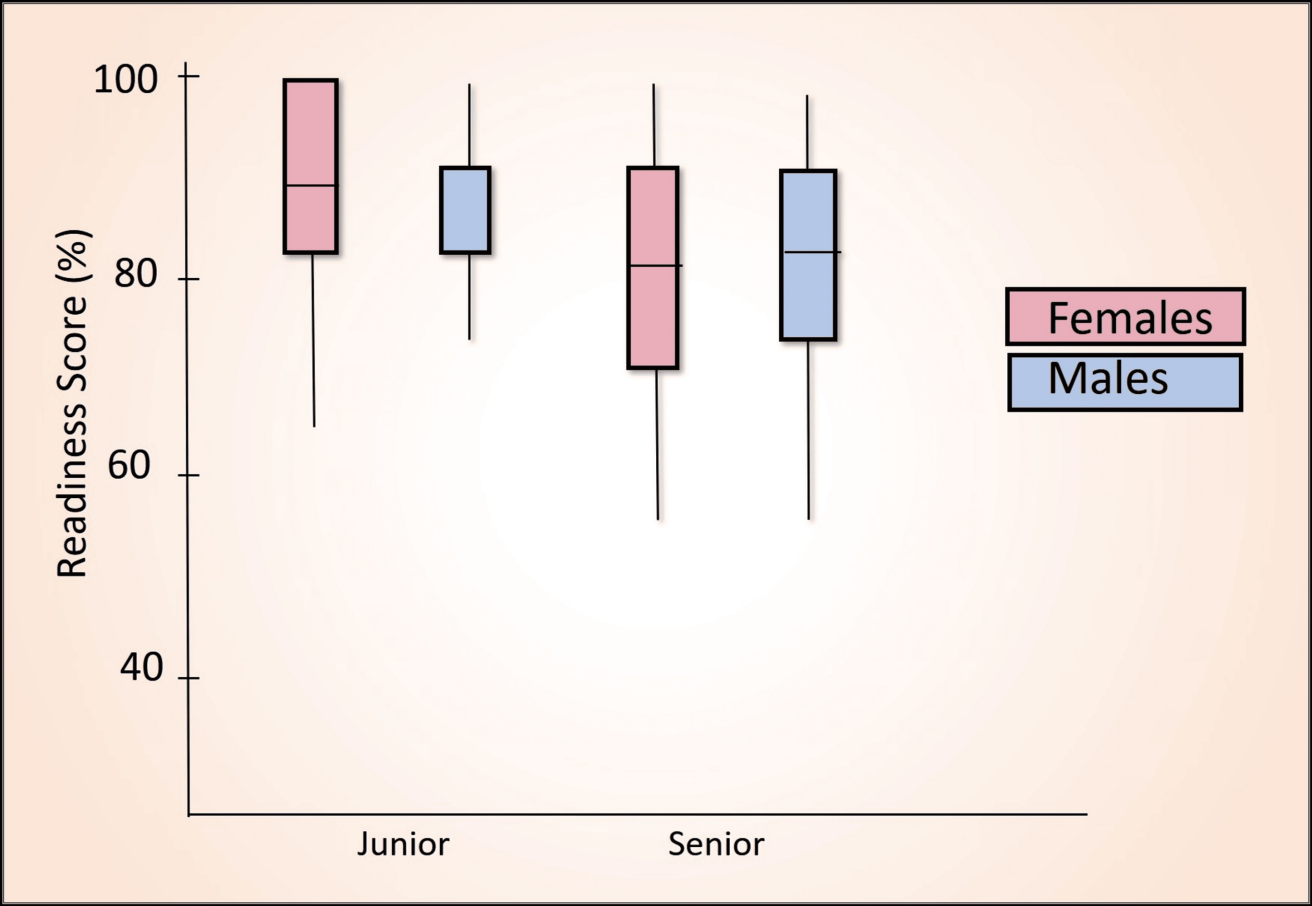
Readiness score (%) comparison by batch and gender

**Figure 6:**
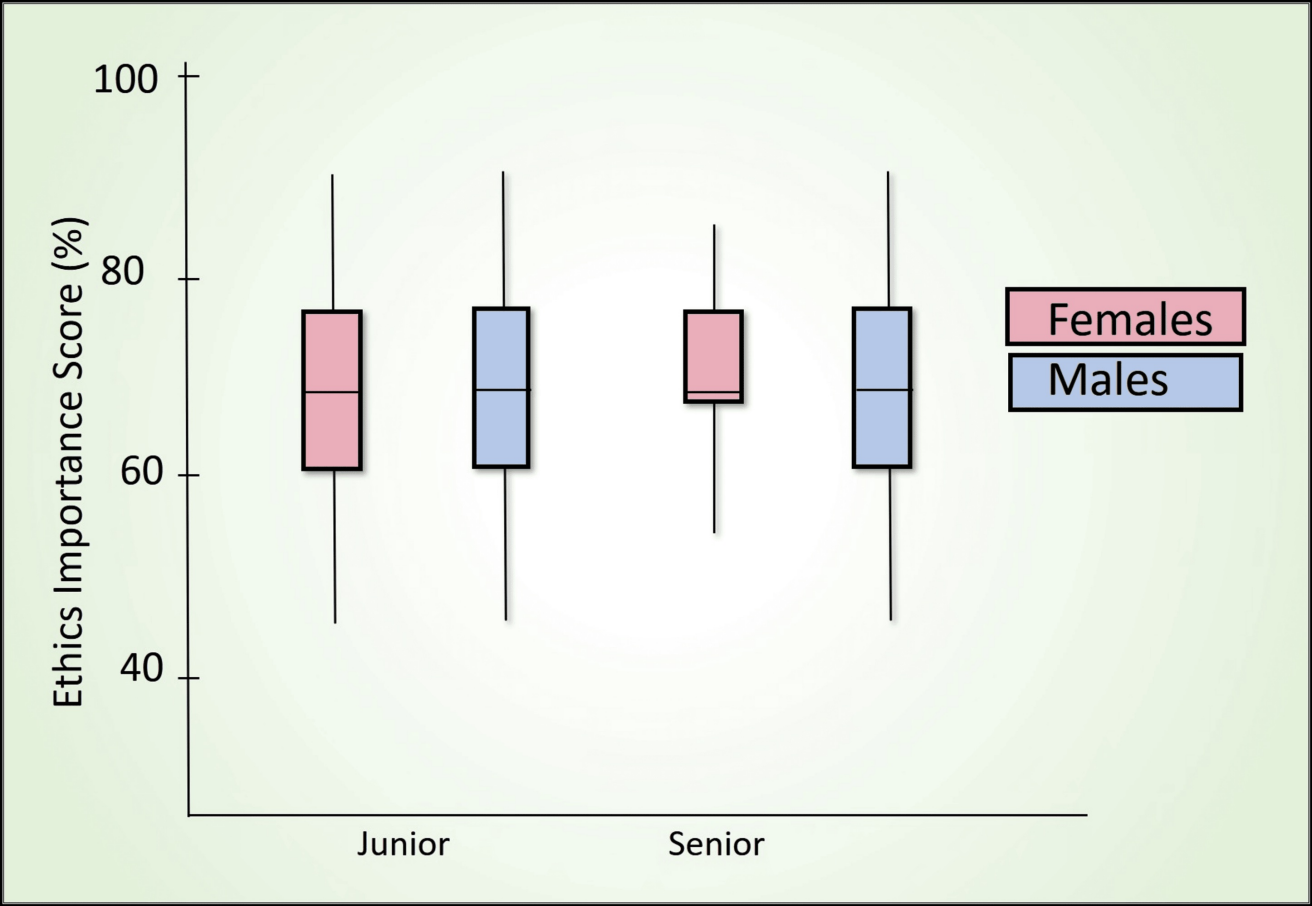
Ethics importance score (%) comparison by batch and gender

Table 7 presents a comparative analysis of the percentage scores between senior and junior batches using the Mann-Whitney U test across various domains, including awareness score (Figure 2), perception score (Figure 3), confidence score (Figure 4), readiness score (Figure 5), and ethics importance score (Figure 6). Specifically, senior batches had slightly higher awareness scores (45.8% versus 43.9%) than junior batches. However, the difference was not statistically significant (Mann-Whitney U = 5487, p = 0.519).

**Table 7:** Comparison of percentage scores between the senior and junior batch *Statistically significant

Variables	Senior batches (n = 94) (2019, 2020, and 2021)		Junior batches (n = 123) (2022 and 2023)		Independent T-test	
	Mean	SD	Mean	SD	statistics	(Mann-Whitney U) P
Awareness score percentage	45.8	14.7	43.9	12.4	5487	0.519
Perception score in percentage	66.8	12.2	70.7	11.5	4635	0.012*
Confidence score in percentage	55.4	18.4	60.8	19.4	4781	0.027*
Readiness score in percentage	87.4	20.4	94.3	14.1	4744	0.003*
Ethics importance score in percentage	68.1	13.9	70.2	12.9	5401	0.399

In contrast, junior batches scored significantly higher in perception (70.7% versus 66.8%, Mann-Whitney U = 4635, p = 0.012), confidence (60.8% versus 55.4%, Mann-Whitney U = 4781, p = 0.027), and readiness (94.3% versus 87.4%, Mann-Whitney U = 4744, p = 0.003).

There was no significant difference in the importance of ethics scores between the senior and junior batches (70.2% versus 68.1%, Mann-Whitney U = 5401, p = 0.399).

In summary, the junior batch outperforms the senior batch in perception, confidence, and readiness scores, with statistically significant differences. In contrast, the scores for the two groups' awareness and importance of ethics do not show significant differences.

## Discussion

The findings from the presented tables shed light on various aspects of medical students' awareness, perceptions, readiness, confidence, and ethical considerations regarding artificial intelligence in healthcare.

Starting with the awareness of AI and its application (Table 1), medical students demonstrated a moderate level of understanding, with an average awareness score of 44.74%. Despite this moderate level, there is room for improvement, particularly in formal education and personal interaction with AI-based healthcare tools, as indicated by the relatively low mean scores in these areas.

Our results are lower than those of the Canadian study by Pucchio et al. [13], which found a 64.3% understanding of AI. Moreover, a German study by McLennan et al. [14] found a 51.6% understanding of AI.

Moving on to perception (Table 2), participants exhibited a generally positive outlook toward AI's role in healthcare, with a mean perception score of 68.96%. This suggests a firm belief in AI's potential to enhance healthcare delivery and improve patient outcomes. However, there is also recognition of the need to understand AI limitations and integrate AI advancements into medical school curricula.

Regarding readiness for AI training (Table 3), medical students expressed a strong willingness to undergo further education and practical training focused on AI in healthcare, with an impressive mean readiness score of 91.32%. This underscores a clear interest among medical students in acquiring the necessary skills to utilize AI technologies in their future medical practice effectively.

Studies by Pucchio et al. [13] and Ejaz et al. [15] reported similar agreement rates, with 78% and 92% of respondents, respectively, sharing this view.

However, despite the high readiness score, there appears to be a moderate confidence level in working with AI applications (Table 4), with a mean confidence score of 58.48%. This suggests that while medical students are eager to undergo training in AI, they may still feel somewhat uncertain about their ability to utilize AI technologies in practice effectively. This highlights a potential area for targeted intervention and support to boost confidence levels.

Finally, medical students deemed ethical considerations surrounding AI in healthcare (Table 5) highly important, with a mean ethics importance score of 69.27%. This underscores the recognition among medical students of the ethical implications associated with AI use in healthcare and the importance of integrating ethical education and training into medical school curricula.

Nine studies emphasize the importance of a basic understanding of AI ethics to promote the adoption of AI in medicine and ensure its safe application [6]. Charow et al. [16] advocated teaching AI-specific ethics in medical schools to address ethical, legal, and data governance challenges.

The findings suggest a strong interest and readiness among medical students to embrace AI in healthcare. However, there are areas, such as confidence in working with AI applications and the need for ongoing ethical education, where further attention and support may be beneficial to ensure the successful integration of AI into medical practice while upholding ethical standards.

The findings from comparing mean scores between male and female medical students reveal exciting insights into the differences. While males tended to have slightly higher scores in awareness, confidence, and readiness, females scored slightly higher in perception and the importance of ethics. However, despite these variations, the statistical analysis showed no significant gender differences in these areas.

These results suggest that both male and female medical students possess relatively similar levels of awareness and understanding of AI in healthcare. However, it is noteworthy that females displayed a slightly higher emphasis on ethical considerations, indicating a potentially greater awareness of the ethical implications associated with AI use in healthcare. This highlights the importance of incorporating ethics education into medical training to ensure that future healthcare professionals, regardless of gender, are equipped to navigate the ethical challenges posed by AI technology.

Furthermore, the comparison between senior and junior batches reveals notable differences. Junior batches, representing more recent cohorts, demonstrated significantly higher scores in perception, confidence, and readiness than senior batches. These findings suggest that newer cohorts of medical students may be more receptive to and prepared for integrating AI technology into healthcare practice. This could be attributed to advancements in medical education curricula, increased exposure to AI technology, and evolving societal attitudes toward healthcare innovation.

Research indicates that machine learning, deep learning, and natural language processing should be integrated into the early stages of undergraduate medical education to enhance students' exposure to artificial intelligence (AI) [16,17].

Interestingly, despite these differences, there were no significant disparities between senior and junior batches regarding awareness and the importance of ethics. This implies that while newer cohorts may be more adept at utilizing AI technology, the foundational understanding of AI and ethical considerations remains consistent across different batches of medical students.

These findings align with a study in Nepal by Jha et al. [11]. Their research reported a median AI knowledge score of 11, with final-year students scoring significantly higher (p = 0.006).

In conclusion, these findings highlight the importance of AI education and ethics training in undergraduate medical curricula to ensure that future healthcare professionals are equipped with the technical skills to utilize AI and a strong understanding of the ethical implications associated with its use.

Additionally, the results highlight the need for ongoing evaluation and adaptation of medical education programs to keep pace with advancements in healthcare technology and societal needs.

Limitation

One limitation of this study is the reliance on self-reported data from medical students, which may introduce response bias and affect the accuracy of the results. Additionally, the study sample may not fully represent the broader population of medical students, potentially limiting the generalizability of the findings. Moreover, the study's cross-sectional design prevents the establishment of causality or the examination of temporal trends over time.

Differences in individual motivation, interest in technology, and career aspirations may influence medical students' engagement with AI education and training opportunities, impacting their AI literacy and readiness levels.

Implication

The implications of this study are multifaceted and relevant for various stakeholders involved in medical education and healthcare.

The findings highlight the importance of integrating AI literacy training into medical school curricula to ensure that future healthcare professionals are adequately prepared to leverage AI technologies in clinical practice. Medical education programs may need to adapt curricula to incorporate AI-related coursework, practical training, and ethical considerations.

Medical schools and institutions may need to invest resources and infrastructure to support AI education and training initiatives. This could include developing AI-specific educational materials, providing access to AI tools and technologies, and offering workshops or training programs focused on AI applications in healthcare.

Continuing education programs and professional development opportunities may be necessary to support current healthcare professionals in enhancing their AI literacy and readiness. Workshops, seminars, and online courses can help practitioners stay abreast of advancements in AI technology and their implications for clinical practice.

Given the importance of ethical considerations in AI adoption, healthcare organizations and regulatory bodies may need to develop guidelines and policies to ensure the ethical use of AI in healthcare. This could include guidelines for data privacy, algorithm transparency, patient consent, and the responsible deployment of AI technologies.

The study underscores the need for further research and innovation in AI education and training within the medical field. Future studies could explore effective teaching methods, evaluate the impact of AI education on clinical outcomes, and assess the long-term implications of AI integration in healthcare delivery.

Overall, the implications of this study emphasize the importance of proactive measures to promote AI literacy, ethical awareness, and readiness among healthcare professionals, ultimately contributing to the practical and responsible adoption of AI technologies in healthcare.

## Conclusions

In summary, this study provides valuable insights into the levels of AI literacy and readiness among future healthcare professionals, as evaluated through comparative analyses of medical students' perceptions, confidence, and ethical considerations. While gender differences did not significantly influence these factors, junior cohorts demonstrated heightened preparedness and receptivity toward AI integration in healthcare compared to their senior counterparts. These findings highlight the importance of fostering AI literacy within medical education curricula in the early years and underscore the necessity for ongoing evaluation and adaptation to ensure that future healthcare professionals are equipped to navigate the complexities of AI in healthcare delivery while upholding ethical standards.

## Appendices

Table 8 presents the questionnaires used in the present study. 

**Table 8:** Students' questionnaires about AI in healthcare and medicine AI: artificial intelligence, ML: machine learning, DL: deep learning

		Questions				Yes		Not sure		No
1a		Have you received any formal education or training on AI in healthcare?								
2a		Have you personally used or interacted with any AI-based healthcare tools or systems?								
3a		Would you be interested in further education or undergraduate training specifically focused on AI in healthcare?								
4a		Do you think practical training with AI tools and systems should be part of undergraduate medical education?								
5a		Do you believe that education and training on ethical considerations of AI should be included in undergraduate medical education?								
	Questions		1	2	3		4		5	
1b	How would you rate your general familiarity with the concept of AI in healthcare?		1. Not Familiar	2. Slightly Familiar	3. Moderately Familiar		4. Familiar		5. Very Familiar	
2b	How would you rate your basic understanding of AI (ML and DL)?		1. Very Limited	2. Limited	3. Moderate		4. Good		5. Very Good	
3b	How would you rate your awareness of AI applications?		1. Not Aware	2. Slightly Aware	3. Moderately Aware		4. Aware		5. Very Aware	
4b	How would you rate your awareness about the use of AI in medicine?		1. Not Aware	2. Slightly Aware	3. Moderately Aware		4. Aware		5. Very Aware	
5b	How would you rate your confidence in working alongside these tools?		1. Not Confident	2. Slightly Confident	3. Moderately Confident		4. Confident		5. Very Confident	
6b	How do you perceive the role of AI in improving healthcare delivery and patient outcomes?		1. No Impact	2. Slight Impact	3. Moderate Impact		4. Significant Impact		5. Very Significant Impact	
7b	Do you believe that AI will significantly impact the future practice of medicine?		1. Strongly Disagree	2. Disagree	3. Neutral		4. Agree		5. Strongly Agree	
8b	How aware are you of the ethical considerations related to the use of AI in healthcare?		1. Not Aware	2. Slightly Aware	3. Moderately Aware		4. Aware		5. Very Aware	
9b	How important do you think it is for medical professionals to consider ethical implications when using AI in healthcare?		1. Not Important	2. Somewhat Important	3. Moderately Important		4. Important		5. Very Important	
10b	How would you rate your understanding regarding the limitations of AI?		1. Very Limited	2. Limited	3. Moderate		4. Good		5. Very Good	
11b	How important do you think it is for medical schools to keep up with advancements in AI and update their curriculum accordingly?		1. Not Important	2. Somewhat Important	3. Moderately Important		4. Important		5. Very Important	
12b	How confident are you in your ability to understand and utilize AI technologies in your future medical practice after obtaining formal training?		1. Not Confident	2. Slightly Confident	3. Moderately Confident		4. Confident 5		5. Very Confident	

## References

[1] Krishnan G, Singh S, Pathania M, Gosavi S, Abhishek S, Parchani A, Dhar M (2023). Artificial intelligence in clinical medicine: catalyzing a sustainable global healthcare paradigm. Front Artif Intell.

[2] Johnson KB, Wei W, Weeraratne D (2020). Precision medicine, AI, and the future of personalized health care. Clin Transl Sci.

[3] Yelne S, Chaudhary M, Dod K, Sayyad A, Sharma R (2023). Harnessing the power of AI: a comprehensive review of its impact and challenges in nursing science and healthcare. Cureus.

[4] Mennella C, Maniscalco U, De Pietro G, Esposito M (2024). Ethical and regulatory challenges of AI technologies in healthcare: a narrative review. Heliyon.

[5] MI MM, MI GM, RA NT (2023). Application of artificial intelligence in medical education: current scenario and future perspectives. J Adv Med Educ Prof.

[6] Pupic N, Ghaffari-zadeh A, Hu R, Singla R, Darras K, Karwowska A, Forster BB (2023). An evidence-based approach to artificial intelligence education for medical students: a systematic review. PLOS Digit Health.

[7] Paranjape K, Schinkel M, Nannan Panday R, Car J, Nanayakkara P (2019). Introducing artificial intelligence training in medical education. JMIR Med Educ.

[8] Wood EA, Ange BL, Miller DD (2021). Are we ready to integrate artificial intelligence literacy into medical school curriculum: students and faculty survey. J Med Educ Curric Dev.

[9] Blease C, Kharko A, Bernstein M (2022). Machine learning in medical education: a survey of the experiences and opinions of medical students in Ireland. BMJ Health Care Inform.

[10] Bisdas S, Topriceanu CC, Zakrzewska Z (2021). Artificial intelligence in medicine: a multinational multi-center survey on the medical and dental students’ perception. Front Public Health.

[11] Jha N, Shankar PR, Al-Betar MA, Mukhia R, Hada K, Palaian S (2022). Undergraduate medical students’ and interns’ knowledge and perception of artificial intelligence in medicine. Adv Med Educ Pract.

[12] Pinto Dos Santos D, Giese D, Brodehl S (2019). Medical students' attitude towards artificial intelligence: a multicentre survey. Eur Radiol.

[13] Pucchio A, Rathagirishnan R, Caton N (2022). Exploration of exposure to artificial intelligence in undergraduate medical education: a Canadian cross-sectional mixed-methods study. BMC Med Educ.

[14] McLennan S, Meyer A, Schreyer K, Buyx A (2022). German medical students´ views regarding artificial intelligence in medicine: a cross-sectional survey. PLOS Digit Health.

[15] Ejaz H, McGrath H, Wong BL, Guise A, Vercauteren T, Shapey J (2022). Artificial intelligence and medical education: a global mixed-methods study of medical students' perspectives. Digit Health.

[16] Charow R, Jeyakumar T, Younus S (2021). Artificial intelligence education programs for health care professionals: scoping review. JMIR Med Educ.

[17] Lee J, Wu AS, Li D, Kulasegaram KM (2021). Artificial intelligence in undergraduate medical education: a scoping review. Acad Med.

